# Environmental Sampling for Avian Influenza A(H7N9) in Live-Poultry Markets in Guangdong, China

**DOI:** 10.1371/journal.pone.0126335

**Published:** 2015-05-01

**Authors:** Min Kang, Jianfeng He, Tie Song, Shannon Rutherford, Jie Wu, Jinyan Lin, Guofeng Huang, Xiaohua Tan, Haojie Zhong

**Affiliations:** 1 Center for Disease Control and Prevention of Guangdong Province, Guangzhou, People’s Republic of China; 2 Centre for Environment and Population Health, School of Environment, Griffith University, Brisbane, Australia; Centers for Disease Control and Prevention, UNITED STATES

## Abstract

**Background:**

To provide an increased understanding of avian influenza A(H7N9) activity in live-poultry market in space and time and hence improve H7N9 epidemic control, an ongoing environmental sampling program in multiple live-poultry markets across Guangdong, China was conducted during March 2013–June 2014.

**Methods:**

A total of 625 live-poultry markets throughout 21 prefecture areas took part in the study. A total of 10 environmental sites in markets for sampling were identified to represent 4 different poultry-related activity areas. At least 10 environmental samples were collected from each market every month. The real time RT-PCR was performed to detect the avian influenza A(H7N9) virus. Field survey was conducted to investigate the sanitation status of live-poultry markets.

**Results:**

There were 109 human infections with H7N9 avian influenza in Guangdong, of which 37 (34%) died. A total of 18741 environmental swabs were collected and subjected to real-time RT-PCR test, of which 905(4.83%) were found positive for H7N9 virus. There were 201 (32.16%) markets affected by H7N9 in 16 prefecture areas. The detection of H7N9 virus in markets spiked in winter months. 63.33% markets (38/60) had no physical segregation for poultry holding, slaughter or sale zones. Closing live-poultry market significantly decreased the H7N9 detection rate from 14.83% (112/755) to 1.67% (5/300).

**Conclusions:**

This study indicates the importance of live-poultry market surveillance based on environmental sampling for H7N9 Avian Influenza control. Improving live-poultry market management and sanitation and changing consumer practices are critical to reduce the risk of H7N9 infection.

## Introduction

In 2013 and 2014 two epidemic waves of an avian influenza A (H7N9) virus (hereafter "H7N9") affected China. The emerging infectious disease first started in Shanghai and spread to eastern and northern China in spring 2013 [1]. The second wave occurred in the winter months between 2013 and 2014 expanding to southern China [2]. By the end of 2014, a total of 469 H7N9 human cases with 187 deaths had been reported from 15 provinces in China [3].

Most human cases of H7N9 had direct or indirect poultry exposure at live-poultry markets (hereafter “LPMs”), where many environmental samples were found to be positive for H7N9 [4–6]. Case-control studies have shown that visiting a LPM increases the risk of H7N9 infection [7]. Live poultry markets have previously been shown to be a common site for avian influenza exposure and infection, notably in the persistence and spread of highly pathogenic avian influenza H5N1 [8,9]. Poultry trades contribute to the H7N9 virus spreading [4,10]. The Chinese Ministry of Agriculture found few H7N9 outbreaks in poultry and none of the birds that tested positive showed clinical disease [11]. Because of its low pathogenicity in poultry, the largely silent infection of H7N9 in poultry is difficult to detect. It is suggested that LPMs, rather than birds or humans, is a better unit for surveillance and analyses [12,13].

In addition to outbreak investigations and virus surveillance in humans, poultry and farms, environmental surveillance in LPMs can improve avian influenza control. Previous studies on H5N1 and H9N2 avian influenza have shown that regular environmental sampling from LPMs can help trace the virus activity and evaluate the risk of human outbreaks [14–16]. To respond to H7N9 outbreaks, many field investigation and studies were performed in LPMs. Poultry samples and environmental swabs were found positive for H7N9 virus. The H7N9-positive rates in LPM environment of eastern China were about 20%-40% [5–7,17]. These findings were mostly based on cross-sectional and retrospective studies. In order to better monitor virus activity and evaluate the risk of human infection with H7N9 virus, continuous environmental surveillance in LPMs is necessary.

Guangdong Province, located in southern China with a population over 100 million, was the epicenter of the second wave of the H7N9 outbreak in 2013–2014 where 109 human cases were reported till June 2014 [18]. To provide an increased understanding of virus activity in the key LPM “reservoirs” in space and time and hence improve H7N9 epidemic control, an ongoing environmental sampling program in multiple LPMs across Guangdong has been initiated since April 2013. This study presents the results of the program in 2013–2014 and discusses the implications for H7N9 prevention and control.

## Materials and Methods

### Human case reporting

Human infection with H7N9 is one of notifiable infectious diseases in China. Case definitions, surveillance for identification of infection, and laboratory test assays have been described previously [1]. All laboratory-confirmed H7N9 human cases in Guangdong are reported to Guangdong Provincial Center for Disease Control and Prevention (hereafter “Guangdong CDC”). Demographic, epidemiological and basic clinical data on each H7N9 case are uploaded to a national case reporting system.

### Expanded LPM surveillance network

Guangdong CDC has conducted LPM surveillance since 2009. 4 prefectures out of 21 prefectures in the province were selected as sentinel sites where environmental specimens were collected and tested monthly from a total of 17 live poultry markets.

Following the first detection of human infection with H7N9 in Shanghai March 2013, Guangdong CDC retrospectively tested environmental specimens collected from sentinel sites for the January to March 2013 period. Then the environmental sampling program for H7N9 expanded to the entire province, such that for each of the 21 prefectures, at least one local LPM was selected on the basis of their poultry sales and market coverage. There were two types of LPM included. One is the wholesale market where live poultry are assembled and held for sale and slaughter. Another is “wet market” retailing live poultry and fresh meat. A total of 625 LPMs, including 21 wholesale markets and 604 wet markets, throughout 21 prefecture cities took part in the study.

### Sample collection and laboratory tests

By field observation and literature review, we identified 10 locations across 4 key activity areas within LMP for swab sampling in this study. The sampling locations include poultry feces, poultry cage, poultry-feeding trough, floor, poultry de-feather machine, Tub, Chopping board, display table, sewage and waste bin. The market activity areas included the poultry-holding zone, the slaughter zone, the sale zone and the waste-disposal zone. Guangdong CDC and 21 prefecture-level CDCs did environmental sampling by collecting at least 10 wet-swab specimens from sampling locations at each prefectures per month. In addition to the planned monitoring across the province, two-week emergence surveillance initiated to collected environmental swabs weekly from LPMs, which were suspected to be epidemiologically associated with human H7N9 infection. As infrastructure and processes differ between LPMs, not all locations were sampled for every market.

A total of 22 national influenza collaborative laboratories located in 21 prefectures participated in this study. All swab samples were sent to the nearest laboratory for detection of H7N9 viral RNA by real-time RT-PCR method [19]. All positive samples were further verified by Guangdong CDC. Sample collection, storage, transportation and laboratory test were based on techniques used in a previous study [20]. All sampling data, including geographic location of each market, sampling date, swab sites, sample types, and laboratory testing results were uploaded to a web-based surveillance information system.

### Field survey for LPM cleaning practices

To access the poultry-related infrastructure and sanitation status in LPMs, we conducted a field survey in January 2014. 60 markets were randomly selected from the surveillance LPMs in Guangzhou, Shenzhen, Dongguan, Foshan and Yangjiang City. We developed a structured questionnaire to investigate each participating market by visual inspection and interviews with managers and vendors. The questionnaire included information about the demarcation of workflow including poultry holding, slaughter, sale, waste-disposal and workplace cleaning practices.

### Data management and analysis

Our study period is from January 2013 to June 2014. A dataset was created containing information on human H7N9 cases and LPMs sampled. The addresses of human H7N9 cases and LPMs were located and geocoded through an Internet geocoding service (http://api.map.baidu.com/lbsapi/getpoint/index.html). We analyzed the dataset and described the temporal and spatial trends by mapping the distribution of H7N9 incidence and affected markets. Chi-squared test (α = 0.05) and Fisher exact test were used to compare the H7N9-positive rate before and after LPM closure.

### Ethics statement

This study was reviewed and approved by the Health Research Ethics committee of Guangdong CDC. Permission was obtained from LPM managers before their participation in the study. No information about the identity of any patients was retained.

## Results

### Human infection

**Fig 1:**
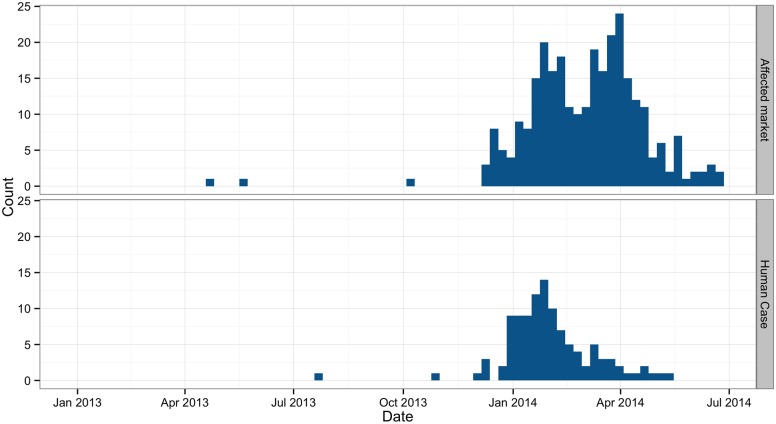
The weekly numbers of H7N9 confirmed human cases (upper) and affected LPMs (lower) in Guangdong, China from January 2013 to June 2014.

**Fig 2:**
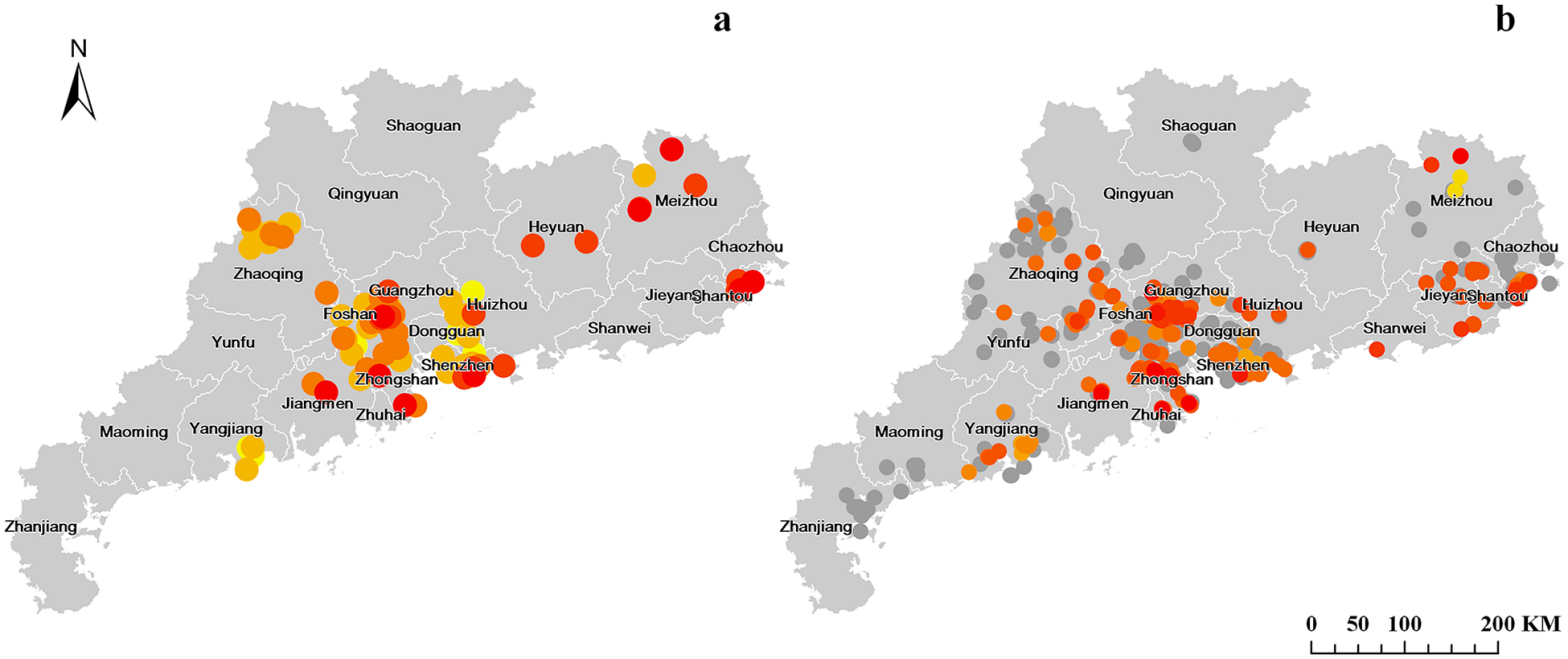
Geographic distribution of H7N9 human infection and H7N9-positive markets in Guangdong. **(a) Human infection with H7N9 in Guangdong. (b) H7N9-positive markets in Guangdong.** In Fig 2a, human cases are shown by coloured points, with colours denoting the chronological order of case detection. Colours range from yellow (earliest cases 28 July 2013) through light and dark orange to red (most recent cases 25 May 2014). The distribution of markets sampled is shown by grey points in Fig 2b. Positive markets are shown by coloured points, with colours denoting the chronological order of detection of affected markets. The yellow points mark the earliest contaminated markets (detection in April and May 2013). The red ones show the latest contaminated markets (detection in June 2014).

By the end of June 2014, 109 people have contracted H7N9 avian influenza, of which 98 (89.91%) had serious disease with pneumonia and 37 (33.94%) died. There were 69 male and 40 female H7N9 cases. The median age of the cases was 56 years (range 2.5–88 years) and 65 (59.63%) occurred in persons aged over 50 years old. The first H7N9 case had an onset of symptoms on July 28 and was confirmed on August 10 2013. Most cases (85 Confirmed, 77.98%) appeared in winter months since December 2013 and the incidence peaked in February 2014. The last H7N9 case in the study period was confirmed at the end of May 2014 (Fig 1). Confirmed cases were identified in 14 of 21 prefectures in Guangdong (Fig 2a). Among the 109 cases, 103 cases (94.50%) reported a history of recent exposure to poultry; of them, 7 cases (6.80%) were poultry-related workers, 16 (15.53%) raised chickens in their household, 25 (24.27%) purchased poultry in markets and 55 (53.40%) had visited LPMs but with no direct contact with poultry before their onset. No possible exposure could be identified for 6 cases.

### LPM contamination

Of 625 LPMs involved, 600 (96.00%) were located in urban areas and 25(4.00%) in rural areas. There were 201 (32.16%) LPMs affected by H7N9 in 16 prefecture areas (Fig 2b). Of them, 158 contaminated markets (78.61%) were located in Pearl River delta areas (Guangzhou, Shenzhen, Dongguan, Foshan, Jiangmen, Zhongshan, Zhuhai). No H7N9 contaminated LPMs were found in the northern prefectures (Qingyuan and Shaoguan) and western prefectures (Maoming and Zhanjiang).

The first two environmental swabs positive for H7N9 virus were found in April and May 2013. They were collected respectively from two LPMs both in Meizhou, a prefecture located in the east of Guangdong. Then a neighboring prefecture Huizhou reported the first human H7N9 case in August 2013. After a comparatively “silent” period, Shenzhen identified a LPM with H7N9 contamination in late October, followed by a H7N9 case reported one week later in a neighboring city, Dongguan. More and more H7N9-positive LPMs and human infection had been identified since December 2013. The affected areas expanded to southern and western cities. Overall, 20 LPMs had been contaminated by H7N9 in 2013. The weekly detection of H7N9-positive LPMs increased sharply at the beginning of 2014 and reached its peak in March. Then it started to decline since April. Between January and June 2014, a total of 195 LPMs had been contaminated by H7N9.

To response to outbreaks of human infection with H7N9, we took emergence surveillance by collecting environmental samples from epidemiologically associated LPMs. Case investigation determined that 87 local H7N9 cases had ever work or visit LPMs before their onset. Additionally, two cases reported in Hong Kong, China, which is closed to Guangdong, had a history of exposure to poultry in LPMs of Guangdong [21]. A total of 99 markets were identified to have epidemiological links to these human infections, of which 44 LPMs (44.44%) had evidence of H7N9 contamination.

A total of 18741 environmental swabs were collected and subjected to real-time RT-PCR test, of which 905(4.83%) were found positive for H7N9 virus. In 2013, the H7N9-positive rate of environmental samples was 0.71 (44/6189). Between January and June 2014, the positive rate was up to 6.86 (861/12552). All four working zones and 8 of 10 sampling sites in LPMs had evidence of H7N9 contamination. The H7N9-positive rates differed significantly in working zones (*χ^2^ = 92.45*, *P<*0.01). The most common affected sites were sewage (10.07%), poultry-feeding trough (9.88%), and chopping board and knife (8.13%). We did not find any H7N9-positive samples in the holding tubs or waste bins (Table 1).

**Table 1:** Results of Real-time RT-PCR Testing for H7N9 in Environmental Samples from Live-poultry Markets, Guangdong, China, January 2013 to June 2014.

Working zone	Sampling site	No. tested	No. positive	Detection rate (%)
Holding	Poultry feces	6535	282	4.32
	Poultry cage	5855	174	2.97
	Poultry-feeding trough	688	68	9.88
	**Subtotal**	**13078**	**524**	**4.01**
Slaughter	Floor	1298	52	4.01
	Poultry de-feathering machine	373	26	6.97
	Tub holding poultry body	81	0	0
	**Subtotal**	**1752**	**78**	**4.45**
Sale	Chopping board & knife	2288	186	8.13
	Display Table	648	44	6.79
	**Subtotal**	**2936**	**230**	**7.83**
Waste disposal	Sewage	725	73	10.07
	Waste bin	250	0	0.00
	**Subtotal**	**975**	**73**	**7.49**
**Total**		**18741**	**905**	**4.83**

### LPM sanitation and impact on closure practices

We conducted a field survey in 60 LPMs of 5 cities (Guangzhou, Foshan, Shenzhen, Dongguan and Yangjiang). All LPMs operated daily, the same vendors operated in the same stalls and the markets had segregated zones for waste disposal. It is shown that 38 markets (63.33%) have no physical segregation for poultry holding, slaughter or sale zones. Live poultry were generally kept in the market for a few days until sold, housing them overnight in stalls. Of 37 LPMs investigated, 24 (64.86%) markets had daily stall washing and 13 (35.14%) disinfected weekly.

Following detection of human infection, some prefecture areas closed local LPMs for two weeks and conducted cleaning and disinfection works in the markets. In 31 LPMs of 4 prefecture areas (Guangzhou, Dongguan, Zhaoqing and Foshan), 755 environmental swabs were sampled in the week before the market closure. 112 samples from 17 markets were H7N9-positive. The H7N9-positive rate was 14.83%. When these LPMs reopened, 300 environmental swabs were collected from the same 31 LPMs. Only 5 samples from 2 markets were found positive for H7N9 and the positive-rate significantly decreased to 1.67% (*χ* ^*2*^ = 37.75, *P*<0.01) (Table 2).

**Table 2:** H7N9-positive Rate of Environmental Samples in Live-poultry Markets, Guangdong, China, Before and After Market Closing and Cleaning.

Areas	Markets sampled	Before Closing and Cleaning		After Closing and Cleaning		*P-value*
		Markets contaminated	Positive samples/tested (%)	Markets contaminated	Positive samples/tested (%)	
Guangzhou	5	5	36/275(13.09)	2	5/137(3.65)	<0.01
Dongguan	10	3	5/84(5.81)	0	0/61(0)	0.06
Zhaoqing	10	7	66/181(36.46)	0	0/64(0)	<0.01
Foshan	6	2	5/215(2.33)	0	0/38(0)	0.44
Total	31	17	112/755(14.83)	2	5/300(1.67)	<0.01

## Discussion

Our study provides baseline information for H7N9 contamination in live poultry markets of Guangdong, south China. With continuing environmental sampling throughout the H7N9 epidemic period, we have detected the importation of H7N9 and traced its time-space changes in LPMs. Our results indicate the H7N9 virus spread across Guangdong with a seasonal peak in winter months.

Unlike other affected areas, Guangdong identified the H7N9 virus in local LPMs environment prior to the detection of human infection. Sequence analyses showed that the Guangdong H7N9 virus isolated from LPM shared a high sequence similarity (98.2% to 99.7%) with other strains from eastern China [20]. First detection in LPMs and virological findings suggest that the viruses isolated from the Guangdong LPM environments were most probably transferred from eastern China, following the first epidemic in eastern China. The interprovincial trade of poultry likely played a key role in transporting H7N9 virus over long distances. Because live poultry industry is large in Guangdong and the business involves importing large quantities of live birds from other areas through marketing channels. Trade routes of poultry from eastern China to Guangdong went across eastern prefectures where H7N9 was brought into and first detected.

Even though Guangdong enhanced human and environmental surveillance, further LPM contamination or human infection was not identified until three months later. It is unclear why H7N9 did not spread quickly in Guangdong following its initial detection. One explanation is that the imported viruses had limited viability within Guangdong poultry and they needed time to adapt to the local conditions. The introduction of H7N9 virus into Guangdong occurred at the end of spring 2013 when the first epidemic wave in China was ending. Before the second wave occurred in winter months, the H7N9 virus may have been circulating in poultries and markets at a low level that was difficult to detect. Another explanation could be the seasonality of H7N9 activity. The sharp increases in winter observed for human H7N9 cases and positive environmental specimens suggest that H7N9 avian influenza follows a similar seasonal pattern as H5N1 avian influenza [22]. Higher consumption of poultry during the days of the Chinese spring festival increased the risk of avian influenza infection [23]. Considering weather factors, the environmental sampling in future should collect meteorological data in the markets, such as air temperature and relative humidity to better understand the link in virus presence and ambient climate conditions.

We use the number of affected markets, rather than data on H7N9-positive samples, as an indicator to provide market-level analysis on the H7N9 contamination level in LPM environment and its temporal and geographical changes. Our figures show a close time-space association between human infection and LPM contamination of H7N9. Contaminated markets were found in most areas where human cases were reported. Our data provide ecological evidence that LPMs are the main source of H7N9 infection. The H7N9 epidemic started in eastern areas of the province and then expanded to southern and western areas. With H7N9 spreading, more and more human cases and contaminated LPMs appeared in the Pearl River delta region, the central part of the province. Given that H7N9 infection has not yet shown continuing person-to-person transmission, poultry trades and consumption contributed to the geographical distribution of H7N9 outbreaks. Because of high density of LPM and more connection between human and poultry, the Pearl River delta has always been referred as a high-risk region for avian influenza epidemics [24]. A study in 2006 shown that 80% respondents in Guangzhou, the capital city of Guangdong Province located in the Pearl River delta, reported an average of 20.5 live chicken purchases annually [25]. A telephone survey conducted by Guangdong CDC in 2014 (unpublished) also show that 68% of families (225/331) in Guangzhou went shopping in wet markets once per week even during the H7N9 epidemic period.

Birds infected with H7N9 normally have mild symptoms, but they can shed the virus for 7 to 10 days [26]. We observed that vendors generally kept live poultry in the market for a few days until sold, housing them overnight in cages. These live poultry staying at markets can get H7N9 infection easily and keep contaminating the market environment persistently. Our findings show that poultry excretions in LPMs contained H7N9 virus and environmental contamination with H7N9 virus was common within LPMs. It is worthy to note that we found H7N9-positive samples in de-feathering machines and chopping tools. These homemade facilities are common used in LPMs for individual slaughtering, which can generate droplets that may contain viral particles and provide high risk for both poultry-related workers and customers in LPMs. Moreover, wet markets usually had no physical segregation for poultry holding, slaughter and sale zones, which are completely open to the costumers. Even if they have no direct contact with live poultry, there is still high potential exposure to the contaminated environment when shopping at wet markets.

Our study shows that closing LPMs reduce H7N9 contamination. It has been already proved that closure of LPMs had indeed decreased the incidence of H7N9 human infection [27]. However its effect on outbreak control may be not sustainable [28]. Shutting LPMs permanently is impractical and creates economic problems. Comprehensive measures should be implemented to prevent and control H7N9 infection. It is necessary to replace individual slaughtering by integrated processing plants and to promote processed poultry products. Regular rest days with no sales of live poultry, a ban on keeping live poultry overnight and enhanced disinfection are proven as effective options [29,30]. More research on the best combination of long-term and short-term measures following detection should be undertaken with a view to reducing H7N9 infection within LPMs.

## Conclusions

This study clearly indicates the importance of environmental sampling in LPMs for H7N9 prevention and control as it can be used to timely monitor the circulation of H7N9 virus and get a better understanding of any H7N9 epidemic. Improving LPM management and sanitation and changing consumer practices are critical to reduce the risk of H7N9 infection.

## Supporting Information

S1 Table Locations of LPMs in Guangdong Province for Environmental Sampling. (DOCX)
