# Supplementary material for: Environmental Sampling for Avian Influenza A(H7N9) in Live-Poultry Markets in Guangdong, China
Source: PLoS One. 2015 May 1;10(5):e0126335. doi: 10.1371/journal.pone.0126335 (PMC4416787; doi:10.1371/journal.pone.0126335)
Supplement: S1 Table — (DOCX) [file pone.0126335.s001.docx]

Table Locations of LPMs in Guangdong Province for Environmental Sampling

| **n** | **Prefectures** | **LPMs** | **Latitude** | **Longitude** |
| --- | --- | --- | --- | --- |
| 1 | Chaozhou | Chao An Qu Jin Shi Shi Chang | 23.53140 | 116.63152 |
| 2 | Chaozhou | Chao An Qu Wu Yang Shi Chang | 23.61298 | 116.60653 |
| 3 | Chaozhou | Chao An Qu Zuo Bu Shi Ji Jia Yuan | 23.46964 | 116.68645 |
| 4 | Chaozhou | Chao An Qu Zuo Bu Zhong Shan Shi Chang | 23.47074 | 116.69160 |
| 5 | Chaozhou | Chao An Xian Gu Xiang Zhen Gu De Jie Shi Chang | 23.66892 | 116.57905 |
| 6 | Chaozhou | Feng Xi Qu Gong Qian Shi Chang | 23.65813 | 116.60743 |
| 7 | Chaozhou | Rao Ping Xian Bei Men Shi Chang | 23.68333 | 117.01201 |
| 8 | Chaozhou | Rao Ping Xian Dong Men Shi Chang | 23.61229 | 116.60679 |
| 9 | Chaozhou | Rao Ping Xian Huang Gang Dong Men Shi Chang | 23.67477 | 117.01079 |
| 10 | Chaozhou | Xiang Qiao Qu Bei Men Shi Chang | 23.68012 | 116.65471 |
| 11 | Chaozhou | Xiang Qiao Qu Lv Zuo Shi Chang | 23.67765 | 116.62938 |
| 12 | Chaozhou | Xiang Qiao Qu Xi Men Shi Chang | 23.66804 | 116.64272 |
| 13 | Chaozhou | Xiang Qiao Qu Xi Zuo Shi Chang | 23.67143 | 116.64860 |
| 14 | Chaozhou | Xiang Qiao Qu Xin Chun Yuan Shi Chang | 23.65638 | 116.63809 |
| 15 | Dongguan | Bai Di Shi Chang | 22.92349 | 114.09246 |
| 16 | Dongguan | Bai Guo Dong Shi Chang | 22.91460 | 114.05121 |
| 17 | Dongguan | Bai Shun Shi Chang | 22.99437 | 113.95328 |
| 18 | Dongguan | Chang Ping Di Yi Rou Cai Shi Chang | 22.97171 | 113.99760 |
| 19 | Dongguan | Chang Ping Lang Bei San Niao Pi Fa Shi Chang | 22.96327 | 114.00727 |
| 20 | Dongguan | Dao Ba Hui Nong Pi Shi | 23.00034 | 113.65680 |
| 21 | Dongguan | Dong Cheng Niu Shan San Niao Pi Fa Shi Chang | 22.94748 | 113.78848 |
| 22 | Dongguan | Dong Cheng San Niao Shi Chang | 23.03085 | 113.77945 |
| 23 | Dongguan | Feng Gang Bu Xin Shi Chang | 22.71389 | 114.16628 |
| 24 | Dongguan | Feng Gang Da Long Gong Ye Qu Shi Chang | 22.72478 | 114.19359 |
| 25 | Dongguan | Feng Gang Feng De Ling | 22.73527 | 114.17227 |
| 26 | Dongguan | Feng Gang Guan Jing Tou Shi Chang | 22.72896 | 114.19631 |
| 27 | Dongguan | Feng Gang Gui Sheng Shu Cai Liang You Dian | 22.75271 | 114.14334 |
| 28 | Dongguan | Feng Gang Hong Ying Shi Chang | 22.72025 | 114.15603 |
| 29 | Dongguan | Feng Gang Huang Dong Shi Chang | 22.75514 | 114.16951 |
| 30 | Dongguan | Feng Gang Jin Feng Huang Shi Chang | 22.74820 | 114.17764 |
| 31 | Dongguan | Feng Gang Nan An Shi Chang | 22.72179 | 114.18917 |
| 32 | Dongguan | Feng Gang Nong Mao Zong He Shi Chang | 22.74383 | 114.14105 |
| 33 | Dongguan | Feng Gang Tang Li Shi Chang | 22.71384 | 114.16753 |
| 34 | Dongguan | Feng Gang Tian Tang Wei | 22.74434 | 114.12472 |
| 35 | Dongguan | Feng Gang Wei De Tian Shi Chang | 22.73004 | 114.19649 |
| 36 | Dongguan | Feng Gang Yan Tian Di San Nong Mao Zong Shi Chang | 22.70582 | 114.18558 |
| 37 | Dongguan | Feng Gang Yan Tian Shi Chang | 22.70390 | 114.17266 |
| 38 | Dongguan | Feng Gang You Gan Pu Shi Chang | 22.71914 | 114.17735 |
| 39 | Dongguan | Feng Gang Zhong Xin Shi Chang | 22.84205 | 114.16940 |
| 40 | Dongguan | Feng Gang Zhu Wei Tian Shi Chang | 22.76700 | 114.13412 |
| 41 | Dongguan | Jin He Zong He Shi Chang | 22.92954 | 114.11886 |
| 42 | Dongguan | Nan Cheng Shi Chang | 23.00653 | 113.73624 |
| 43 | Dongguan | Qiao Tou Zhen San Niao Shi Chang | 23.03335 | 114.08014 |
| 44 | Dongguan | San Yong San Niao Shi Chang | 23.13222 | 113.71831 |
| 45 | Dongguan | Sha Hu Shi Chang | 22.77257 | 114.07368 |
| 46 | Dongguan | Shi Ma Shi Chang | 22.90857 | 114.08559 |
| 47 | Dongguan | Shi Ma Zong He Shi Chang | 22.91826 | 114.09497 |
| 48 | Dongguan | Yin Ma Shi Chang | 22.90738 | 114.09574 |
| 49 | Dongguan | Yuan Shan Bei Shi Chang | 22.95960 | 113.98240 |
| 50 | Dongguan | Zhang Luo Shi Chang | 22.91146 | 114.07274 |
| 51 | Dongguan | Zhang Mu Tou Zong He Shi Chang | 22.90878 | 114.07741 |
| 52 | Dongguan | Zhang Yang Shi Chang | 22.88015 | 114.09447 |
| 53 | Dongguan | Zhen Nan Cheng Shi Chang | 22.79323 | 113.69657 |
| 54 | Dongguan | Zhong Tang San Niao Shi Chang | 23.09707 | 113.66334 |
| 55 | Dongguan | Zhong Xin Shi Chang | 22.72077 | 114.17261 |
| 56 | Dongguan | Zuo Cheng Jin Sha Shi Chang | 23.04854 | 113.77642 |
| 57 | Foshan | Bei Zhen Gao Cun Shi Chang | 22.95394 | 113.20271 |
| 58 | Foshan | Bei Zhen He Cheng Nong Mao Shi Chang | 22.95997 | 113.23702 |
| 59 | Foshan | Chen Cun Zhen Jiu Zuo Shi Chang | 22.96770 | 113.23017 |
| 60 | Foshan | Chen Cun Zhen Xin Zuo Shi Chang | 22.96514 | 113.25386 |
| 61 | Foshan | Da Li Gui Jiang San Niao Shi Chang | 23.13156 | 113.11943 |
| 62 | Foshan | Da Liang Jie Dao Guan Guang Shi Chang | 22.85643 | 113.25420 |
| 63 | Foshan | De Bao Shi Chang | 23.17980 | 112.89492 |
| 64 | Foshan | Gao Ming Qu He Cheng Shi Chang | 22.90132 | 112.88708 |
| 65 | Foshan | Gao Ming Qu Wen Hua Shi Chang | 22.89888 | 112.90082 |
| 66 | Foshan | Guan Yao Shi Chang | 23.23281 | 113.09235 |
| 67 | Foshan | Gui Cheng Nan Xing Shi Chang | 23.03479 | 113.15610 |
| 68 | Foshan | Gui Hua Shi Chang | 23.03712 | 113.14523 |
| 69 | Foshan | Huang Zuo Shi Chang | 23.12153 | 113.19612 |
| 70 | Foshan | Huang Zuo Yong Sheng Shi Chang | 23.11731 | 113.20438 |
| 71 | Foshan | Jiu Jiang Yi Feng Shi Chang | 22.83733 | 113.02276 |
| 72 | Foshan | Jun An Zhen Bai An Xian Dai Shi Chang | 22.72359 | 113.15668 |
| 73 | Foshan | Jun An Zhen Zhong Xin Shi Chang | 22.70859 | 113.16281 |
| 74 | Foshan | Le Cong Le Tai Ju Shi Chang | 22.96683 | 113.10749 |
| 75 | Foshan | Le Cong Zhen Teng Chong Shi Chang | 22.96674 | 113.10762 |
| 76 | Foshan | Le Liu Jie Dao Guang Da Shi Chang | 22.86396 | 113.13990 |
| 77 | Foshan | Le Liu Zhen Yin Cheng Shi Chang | 22.86428 | 113.13980 |
| 78 | Foshan | Li Shui Sha Yong Zong He Shi Chang | 23.14828 | 113.17123 |
| 79 | Foshan | Li Shui Zhen Gan Jiao Shi Chang | 23.17906 | 113.15066 |
| 80 | Foshan | Lian Hua Shi Chang | 23.04049 | 113.12091 |
| 81 | Foshan | Long Jiang Zhen Liao Liu Zuo Pi Fa Ji Dang | 22.86767 | 113.07622 |
| 82 | Foshan | Long Jiang Zhen Ying Xin Jie Shi Chang | 22.87926 | 113.08520 |
| 83 | Foshan | Lun Jiao San Zhou Nong Chang Pin Jiao Yi Zhong Xin | 22.89104 | 113.28561 |
| 84 | Foshan | Luo Pu Shi Chang | 22.84224 | 113.01253 |
| 85 | Foshan | Min An Shi Chang | 22.89723 | 112.87094 |
| 86 | Foshan | Nan Hai Qu Bei Yue Shi Chang | 23.04464 | 113.15449 |
| 87 | Foshan | Nan Hai Qu Da Li Zhen Li Xi Da Gang Shi Chang | 23.12759 | 113.12089 |
| 88 | Foshan | Nan Hai Qu Gui Cheng Dong Er Nong Mao Shi Chang | 23.03376 | 113.15696 |
| 89 | Foshan | Gui Cheng Tian You Er Lu Zuo Mu Dan Cun Shi Chang | 23.04289 | 113.14434 |
| 90 | Foshan | Nan Hai Qu Le Qing Zong He Shi Chang | 23.05118 | 113.14159 |
| 91 | Foshan | Nan Yue Shi Chang | 23.04429 | 113.15390 |
| 92 | Foshan | Pu Jun Shi Chang | 23.03046 | 113.13003 |
| 93 | Foshan | Rong Gui Jie Dao An Bian Shi Chang | 22.77690 | 113.29044 |
| 94 | Foshan | Rong Gui Jie Dao Gui Zuo Shi Chang | 22.76433 | 113.26976 |
| 95 | Foshan | Rong Gui Jie Dao Hai Wei Di Er Shi Chang | 22.75450 | 113.27917 |
| 96 | Foshan | Rong Gui Jie Dao Hai Wei Shi Chang | 22.74862 | 113.28701 |
| 97 | Foshan | Rong Gui Jie Dao Shang Jia Shi Nong Mao Shi Chang | 22.76885 | 113.29170 |
| 98 | Foshan | San Shui Qu Xi Nan Bai Wang Cheng Shi Chang | 23.18009 | 112.90832 |
| 99 | Foshan | San Shui Qu Xi Nan Jie Dao Yuan Lin Shi Chang | 23.16832 | 112.89638 |
| 100 | Foshan | San Shui Xi Nan Shang Ye Cheng Shi Chang | 23.17008 | 112.90638 |
| 101 | Foshan | Shi Tou Shi Chang | 22.98665 | 113.12627 |
| 102 | Foshan | Wan Hua Shi Chang | 23.01427 | 113.14754 |
| 103 | Foshan | Wu Yang Shi Chang | 22.89927 | 112.88432 |
| 104 | Foshan | Xi Nan Bu Xin Shi Chang | 23.17799 | 112.87951 |
| 105 | Foshan | Xi Nan Sha Tou Shi Chang | 23.15666 | 112.90494 |
| 106 | Foshan | Xi Nan Shi Chang | 23.16264 | 112.89748 |
| 107 | Foshan | Xi Nan Zhang Bian Shi Chang | 23.17827 | 112.90330 |
| 108 | Foshan | Xing Hua Shi Chang | 22.89665 | 112.88875 |
| 109 | Foshan | Xing Tan Zhen Gao Zan Shi Chang | 22.76936 | 113.20462 |
| 110 | Foshan | Xing Tan Zhen Guang Hui Shi Chang | 22.77955 | 113.18681 |
| 111 | Foshan | Xing Tan Zhen Xing Tan Shi Chang | 22.78603 | 113.16263 |
| 112 | Foshan | Yan Bu Huan Qiu Shi Chang | 23.11600 | 113.16316 |
| 113 | Foshan | Yuan Lin Shi Chang | 23.16864 | 112.89645 |
| 114 | Foshan | Zhang Zuo Shi Chang | 23.04170 | 113.08738 |
| 115 | Foshan | Zhong Nan Nong Chan Pin Jiao Yi Zhong Xin | 23.06922 | 113.10663 |
| 116 | Foshan | Zhou Bian Shi Chang | 23.11728 | 112.86696 |
| 117 | Foshan | Zuo Cheng Qu Hui Jing Shi Chang | 23.00730 | 113.11734 |
| 118 | Foshan | Zuo Cheng Qu Ji Hua Mei Shi Cheng Fu Long Xuan | 23.01816 | 113.10699 |
| 119 | Foshan | Zuo Cheng Qu Liu Yuan Shi Chang | 23.01310 | 113.09627 |
| 120 | Foshan | Zuo Cheng Qu Nan Zhuang Ji Li Shi Chang | 22.97990 | 113.01659 |
| 121 | Foshan | Zuo Cheng Qu Yin Yuan Shi Chang | 22.99110 | 113.11245 |
| 122 | Foshan | Zuo Cheng Qu Zuo Tang Shi Chang | 23.02327 | 113.10125 |
| 123 | Guangdong | Cong Hua Jie Bian Shi Chang | 23.43268 | 113.49229 |
| 124 | Guangzhou | Ba Qi Xin Jie Shi | 23.12255 | 113.28332 |
| 125 | Guangzhou | Bai He Yuan Nong Fu Chan Pin Zong He Shi Chang | 23.09968 | 113.24279 |
| 126 | Guangzhou | Bai Xing San Niao Qin Lei Pi Fa Shi Chang | 23.24470 | 113.31153 |
| 127 | Guangzhou | Bai Yun Xin Jie Shi | 23.12537 | 113.28911 |
| 128 | Guangzhou | Chang Zong He Shi Chang | 23.17376 | 113.36017 |
| 129 | Guangzhou | Chi Gang Rou Cai Shi Chang | 23.09583 | 113.32401 |
| 130 | Guangzhou | Chi Sha Nan Xing Zong He Shi Chang | 23.08937 | 113.36380 |
| 131 | Guangzhou | Cong Hua Xing Fu Shi Chang | 23.43269 | 113.49229 |
| 132 | Guangzhou | Da Lin Gang Shi Chang | 23.13326 | 113.43525 |
| 133 | Guangzhou | Da Men Kou Shi Chang | 23.12295 | 113.56030 |
| 134 | Guangzhou | Da Sha Dong Shi Chang | 23.11130 | 113.45541 |
| 135 | Guangzhou | Da Sha Ji Tang Shi Chang | 23.14238 | 113.47346 |
| 136 | Guangzhou | Deng Feng Rou Cai Shi Chang | 23.13857 | 113.27776 |
| 137 | Guangzhou | Dong Cheng Shi Chang | 22.94176 | 113.37791 |
| 138 | Guangzhou | Dong Fang Xin Jie Shi | 23.10940 | 113.47510 |
| 139 | Guangzhou | Dong Hu Xin Cun Shi Chang | 23.12272 | 113.29264 |
| 140 | Guangzhou | Dong Ji Shi Chang | 23.07008 | 113.52080 |
| 141 | Guangzhou | Dong Jun Shi Chang | 23.08692 | 113.34319 |
| 142 | Guangzhou | Dong Pu Nong Mao Zong He Shi Chang | 23.12361 | 113.40630 |
| 143 | Guangzhou | Dong Pu Zhu Cun Zong He Shi Chang | 23.12056 | 113.42597 |
| 144 | Guangzhou | Dong Qu Nong Mao Shi Chang | 23.28666 | 113.85093 |
| 145 | Guangzhou | Dong Yuan Rou Cai Shi Chang | 23.13167 | 113.31115 |
| 146 | Guangzhou | Feng Le Shi Chang | 23.10755 | 113.46244 |
| 147 | Guangzhou | Fu Jin Rou Cai Shi Chang | 23.13723 | 113.30902 |
| 148 | Guangzhou | Fu Li Shi Chang | 23.14734 | 113.23552 |
| 149 | Guangzhou | Gang Wan Bei Shi Chang | 23.11146 | 113.45247 |
| 150 | Guangzhou | Gang Wan Yi Cun Shi Chang | 23.10611 | 113.45182 |
| 151 | Guangzhou | Gao Zeng Nong Mao Shi Chang | 23.35382 | 113.31409 |
| 152 | Guangzhou | Ge Xin Shi Chang | 23.10073 | 113.25843 |
| 153 | Guangzhou | Gong He Nong Mao Shi Chang | 23.13131 | 113.31073 |
| 154 | Guangzhou | Guang Da Rou Cai Shi Chang | 23.13325 | 113.27411 |
| 155 | Guangzhou | Guo Liu Bao Rou Cai Shi Chang | 23.12681 | 113.33709 |
| 156 | Guangzhou | Hai Zhu Nan Shi Chang | 23.09879 | 113.26506 |
| 157 | Guangzhou | Hai Zhu Qu Nan Ji Shi Chang | 23.07568 | 113.27595 |
| 158 | Guangzhou | Hai Zhu Shi Chang | 23.11952 | 113.26343 |
| 159 | Guangzhou | Hao Xian Shi Chang | 23.13623 | 113.27967 |
| 160 | Guangzhou | He Ding Shi Chang | 23.08618 | 113.25094 |
| 161 | Guangzhou | He Dong Qi Xing Shi Chang | 23.54422 | 113.60787 |
| 162 | Guangzhou | He Feng Shi Chang | 23.22103 | 113.56987 |
| 163 | Guangzhou | He Le Shi Chang | 23.29660 | 113.36702 |
| 164 | Guangzhou | Heng Tan Rou Cai Shi Chang | 23.38399 | 113.22218 |
| 165 | Guangzhou | Hua Jing Jing Zuo Shi Chang | 23.14628 | 113.36719 |
| 166 | Guangzhou | Hua Long Shi Chang | 23.13022 | 113.40090 |
| 167 | Guangzhou | Hua Long Zhen Shi Chang | 23.04615 | 113.46294 |
| 168 | Guangzhou | Huang Bian Shi Chang | 22.95651 | 113.37908 |
| 169 | Guangzhou | Huang Cun Rou Cai Zong He Shi Chang | 23.12883 | 113.42177 |
| 170 | Guangzhou | Huang Cun Xin Fu Zong He Shi Chang | 23.12862 | 113.42112 |
| 171 | Guangzhou | Huang Jia Rou Cai Shi Chang | 23.14766 | 113.29225 |
| 172 | Guangzhou | Hui Fu Xin Jie Shi | 23.12661 | 113.26961 |
| 173 | Guangzhou | Ji Shan Dong Shi Chang | 23.13291 | 113.43982 |
| 174 | Guangzhou | Ji Xian Yuan Rou Cai Shi Chang | 23.24067 | 113.30705 |
| 175 | Guangzhou | Jia He Nong Mao Shi Chang | 23.23730 | 113.28113 |
| 176 | Guangzhou | Jian She Xin Cun Shi Chang | 23.14053 | 113.28849 |
| 177 | Guangzhou | Jiang Bao Rou Cai Shi Chang | 23.10906 | 113.27493 |
| 178 | Guangzhou | Jiang Gao Qin Lei Pi Fa Shi Chang | 23.29307 | 113.23467 |
| 179 | Guangzhou | Jiang Pu Jie Qi Xing Rou Cai Shi Chang | 23.54474 | 113.60678 |
| 180 | Guangzhou | Jin Bi Shi Chang | 23.22812 | 113.29709 |
| 181 | Guangzhou | Jin Hua Xin Shi Chang | 23.13342 | 113.26067 |
| 182 | Guangzhou | Jin Sha Zong He Shi Chang | 23.08800 | 113.29276 |
| 183 | Guangzhou | Jin Zhou Shi Chang | 22.80689 | 113.55306 |
| 184 | Guangzhou | Jin Zi Li Zong He Shi Chang | 23.08756 | 113.29259 |
| 185 | Guangzhou | Jiu Fo Feng Huang Shi Chang | 23.35735 | 113.52886 |
| 186 | Guangzhou | Jun Jing Xin Jie Shi | 23.12670 | 113.39221 |
| 187 | Guangzhou | Ke Cun Nong Mao Shi Chang | 23.09819 | 113.33380 |
| 188 | Guangzhou | Ke Shan Zong He Shi Chang | 23.14968 | 113.25416 |
| 189 | Guangzhou | Keng Kou Shi Chang | 23.09473 | 113.24307 |
| 190 | Guangzhou | Lai An Shi Chang | 23.20984 | 113.55610 |
| 191 | Guangzhou | Lai Fu Rou Cai Shi Chang | 23.11491 | 113.28172 |
| 192 | Guangzhou | Li Cheng Jie Fu Peng Shi Chang | 23.30217 | 113.83738 |
| 193 | Guangzhou | Li Cheng Jie Xing Fa Shi Chang | 23.29864 | 113.84060 |
| 194 | Guangzhou | Li Lian Jie Da Men Kou Shi Chang | 23.12272 | 113.56073 |
| 195 | Guangzhou | Li Nong Mao Shi Chang | 23.06856 | 113.32279 |
| 196 | Guangzhou | Li Xiang Shi Chang | 23.10247 | 113.45088 |
| 197 | Guangzhou | Lian He Jie Chang An Shi Chang | 23.19344 | 113.44548 |
| 198 | Guangzhou | Lie De Rou Cai Shi Chang | 23.12054 | 113.34332 |
| 199 | Guangzhou | Lin He Dong Zong He Rou Cai Shi Chang | 23.15432 | 113.33519 |
| 200 | Guangzhou | Liu Hua Xin Jie Shi | 23.14713 | 113.25718 |
| 201 | Guangzhou | Long Dong Nong Mao Shi Chang | 23.20062 | 113.37163 |
| 202 | Guangzhou | Long Dong Shi Chang | 23.19836 | 113.37436 |
| 203 | Guangzhou | Long Jin Shi Chang | 23.12999 | 113.25050 |
| 204 | Guangzhou | Luo Gang Qu Bi Cun Zong He Shi Chang | 23.11048 | 113.53807 |
| 205 | Guangzhou | Luo Gang Qu Hua Long Shi Chang | 23.28361 | 113.59017 |
| 206 | Guangzhou | Luo Gang Qu Hua Ting Shi Chang | 23.15499 | 113.50645 |
| 207 | Guangzhou | Luo Gang Qu Lian He Shi Chang | 23.20584 | 113.43104 |
| 208 | Guangzhou | Luo Gang Shi Chang | 23.18121 | 113.51230 |
| 209 | Guangzhou | Mei Yuan Nan Shi Chang | 23.09883 | 113.26501 |
| 210 | Guangzhou | Mi Shi Xin Jie Shi | 23.12682 | 113.26556 |
| 211 | Guangzhou | Miao Qing Shi Chang | 22.78691 | 113.47875 |
| 212 | Guangzhou | Nan Hua Shi Chang | 23.39257 | 113.22197 |
| 213 | Guangzhou | Nan Sha Jie Ban Tou Shi Chang | 22.79672 | 113.55447 |
| 214 | Guangzhou | Nan Sha Jie Xin Jun Shi Chang | 22.80619 | 113.55534 |
| 215 | Guangzhou | Pan Fu Lan Hu Li Shi Chang | 23.14034 | 113.26460 |
| 216 | Guangzhou | Pu Zuo Shi Chang | 23.05883 | 113.53326 |
| 217 | Guangzhou | Qi Fu Shi Chang | 22.97374 | 113.34294 |
| 218 | Guangzhou | Qian Kou Shi Chang | 23.11491 | 113.26976 |
| 219 | Guangzhou | Qiao Dong Shi Chang | 23.11368 | 113.22155 |
| 220 | Guangzhou | Qiao Tou Rou Cai Shi Chang | 23.14129 | 113.22923 |
| 221 | Guangzhou | Qing He Pi Fa Shi Chang | 22.94076 | 113.40567 |
| 222 | Guangzhou | Qing Nian Lu Shi Chang | 23.07235 | 113.53348 |
| 223 | Guangzhou | Qing Ping Dong Rou Cai Shi Chang | 23.11622 | 113.25339 |
| 224 | Guangzhou | Rui Bao Rou Cai Shi Chang | 23.07383 | 113.30011 |
| 225 | Guangzhou | Sha Dong Shi Chang | 23.16609 | 113.32958 |
| 226 | Guangzhou | Sha He Deng Feng Zong He Shi Chang | 23.14983 | 113.28608 |
| 227 | Guangzhou | Sha Xu Shi Chang | 22.94687 | 113.38610 |
| 228 | Guangzhou | Sha Yong Shi Chang | 23.16777 | 113.26873 |
| 229 | Guangzhou | Sha Yuan Nong Fu Chan Pin Shi Chang | 23.09504 | 113.27099 |
| 230 | Guangzhou | Sha Yuan Shi Chang | 23.09639 | 113.27103 |
| 231 | Guangzhou | Shan Cun Zong He Xin Jie Shi | 23.10997 | 113.23679 |
| 232 | Guangzhou | Shan Ding Shi Chang | 23.07964 | 113.24999 |
| 233 | Guangzhou | Shang She Nong Mao Rou Cai Shi Chang | 23.15104 | 113.35752 |
| 234 | Guangzhou | Shang Yuan Gang Zong He Shi Chang | 23.16749 | 113.33571 |
| 235 | Guangzhou | Shi Ji Zhen Guang Chang Shi Chang | 22.96063 | 113.44887 |
| 236 | Guangzhou | Shi Xi Xin Nong Mao Zong He Shi Chang | 23.11390 | 113.41943 |
| 237 | Guangzhou | Shuang Qiao Rou Cai Shi Chang | 23.12410 | 113.22840 |
| 238 | Guangzhou | Shun Fa Shi Chang | 23.14570 | 113.22641 |
| 239 | Guangzhou | Si She Xin Shi Chang | 23.14463 | 113.22636 |
| 240 | Guangzhou | Si You Shi Chang | 23.12519 | 113.31636 |
| 241 | Guangzhou | Tai He Qin Lei Pi Fa Shi Chang | 23.30645 | 113.35100 |
| 242 | Guangzhou | Tai He Shi Chang | 23.30138 | 113.35880 |
| 243 | Guangzhou | Tai He Xie Jia Zhuang Nong Mao Zong He Shi Chang | 23.30503 | 113.36145 |
| 244 | Guangzhou | Tai Ping Sha Rou Cai Shi Chang | 23.11492 | 113.26976 |
| 245 | Guangzhou | Tai Ping Zhen Rong Biao Shi Chang | 23.43732 | 113.48814 |
| 246 | Guangzhou | Tai Yong Shi Chang | 23.08331 | 113.34133 |
| 247 | Guangzhou | Tan Wei Zhong Xin Shi Chang | 23.12587 | 113.22771 |
| 248 | Guangzhou | Tang De Nong Fu Chan Pin Zong He Shi Chang | 23.14173 | 113.38527 |
| 249 | Guangzhou | Tao Jin Nong Mao Shi Chang | 23.14675 | 113.29690 |
| 250 | Guangzhou | Wu Yan Qiao Rou Cai Shi Chang | 23.10631 | 113.21872 |
| 251 | Guangzhou | Xi Gang Shi Chang | 23.09730 | 113.28801 |
| 252 | Guangzhou | Xi Hua Lu Yi Jin Shi Chang | 23.13730 | 113.25890 |
| 253 | Guangzhou | Xi Hua Shi Chang | 23.09642 | 113.27103 |
| 254 | Guangzhou | Xi Ji Shi Chang | 23.09550 | 113.27153 |
| 255 | Guangzhou | Xie Cun Pi Fa Shi Chang | 22.97380 | 113.31965 |
| 256 | Guangzhou | Xie Min Pi Fa Shi Chang | 23.10301 | 113.52696 |
| 257 | Guangzhou | Xin Tang Zong He Shi Chang | 23.16411 | 113.41813 |
| 258 | Guangzhou | Xin Zong He Shi Chang | 23.09438 | 113.32442 |
| 259 | Guangzhou | Xing Fa Shi Chang | 23.28903 | 113.83684 |
| 260 | Guangzhou | Xing Yun Shi Chang | 23.13151 | 113.28705 |
| 261 | Guangzhou | Ya Yao Rou Cai Shi Chang | 23.36902 | 113.22860 |
| 262 | Guangzhou | Yang Ji Shi Chang | 23.13592 | 113.32479 |
| 263 | Guangzhou | Yao Tai Xing Long Shi Chang | 23.16588 | 113.25935 |
| 264 | Guangzhou | Yong He Jie Gan Zhu Shi Chang | 23.20477 | 113.56699 |
| 265 | Guangzhou | Yong He Jie Heng Zuo Zong He Shi Chang | 23.19338 | 113.57591 |
| 266 | Guangzhou | Yong He Jie Xiao Dong Shi Chang | 23.19623 | 113.56831 |
| 267 | Guangzhou | Yong Sheng Rou Cai Shi Chang | 23.12879 | 113.28564 |
| 268 | Guangzhou | Yu Zhu Di Tie Zhan Jie Shi | 23.10613 | 113.45206 |
| 269 | Guangzhou | Yuan Cun Shi Chang | 23.12727 | 113.36924 |
| 270 | Guangzhou | Yun Yuan Shi Chang | 23.17312 | 113.27572 |
| 271 | Guangzhou | Zeng Cheng Shi Li Cheng Jie Gong Ye Pin Shi Chang | 23.29462 | 113.84248 |
| 272 | Guangzhou | Zhen Long Jiu Shi Chang | 23.28125 | 113.58265 |
| 273 | Guangzhou | Zhi Chang Rou Cai Shi Chang | 23.08024 | 113.27630 |
| 274 | Guangzhou | Zhou Men Shi Chang | 23.13351 | 113.24300 |
| 275 | Guangzhou | Zhu Guang Shi Chang | 23.12432 | 113.27913 |
| 276 | Guangzhou | Zhu Ying Nong Mao Zong He Shi Chang | 23.12039 | 113.42655 |
| 277 | Guangzhou | Zi Lai Rou Cai Shi Chang | 23.12862 | 113.25619 |
| 278 | Guangzhou | Zuo Cun Shi Chang | 23.16549 | 113.38759 |
| 279 | Guangzhou | Zuo Tou Zhen Qi Gan Zong He Shi Chang | 23.58588 | 113.51093 |
| 280 | Guangzhou | Zuo Zuo Shi Chang | 23.15918 | 113.40451 |
| 281 | Guangzhou | Zuo Zuo Shi Shi Chang | 23.12814 | 113.43245 |
| 282 | Heyuan | Shang Jiao Shi Chang | 23.74605 | 114.70140 |
| 283 | Heyuan | Wen Chang Shi Chang | 23.75653 | 114.71541 |
| 284 | Huizhou | Fu Tian Shi Chang | 23.22064 | 113.98415 |
| 285 | Huizhou | Jiang Bei San Niao Pi Fa Shi Chang | 23.13993 | 114.45065 |
| 286 | Huizhou | Jiang Bei San Xin Shi Chang | 23.13051 | 114.42508 |
| 287 | Huizhou | Long Xi Xin Shi Chang | 23.14264 | 114.14132 |
| 288 | Huizhou | Long Xi Zhen Zhong Xin Shi Chang | 23.14286 | 114.14108 |
| 289 | Huizhou | San Xin Ji Mao Shi Chang | 23.13217 | 114.42401 |
| 290 | Huizhou | Shui Bei Shi Chang | 23.11450 | 114.43290 |
| 291 | Huizhou | Wang Jiang Shi Chang | 23.12718 | 114.44312 |
| 292 | Jiangmen | Ba Chong Shi Chang | 22.61282 | 113.08548 |
| 293 | Jiangmen | Bai Sha Jiang Nan Qin Xu Pi Fa Shi Chang | 22.57924 | 113.07102 |
| 294 | Jiangmen | Bei Jiao Shi Chang | 22.61437 | 113.09214 |
| 295 | Jiangmen | Feng Le Shi Chang | 22.61096 | 113.09603 |
| 296 | Jiangmen | Guan Xi Shi Chang | 22.58765 | 113.08836 |
| 297 | Jiangmen | Hui Cheng Ren Yi Shi Chang | 22.51573 | 113.03122 |
| 298 | Jiangmen | Hui Cheng Yue Yang Shi Chang | 22.52440 | 113.05301 |
| 299 | Jiangmen | Kai Ping Shi Lv Huang Jia Qin Pi Fa Shi Chang | 22.39570 | 112.72225 |
| 300 | Jiangmen | Kai Ping Shi Xiang Long Shi Chang | 22.36193 | 112.68321 |
| 301 | Jiangmen | Kai Ping Shi Yin Hai Shi Chang | 22.37993 | 112.71433 |
| 302 | Jiangmen | Li Le Wen Chang Shi Chang | 22.55893 | 113.08961 |
| 303 | Jiangmen | Mei Jing Shi Chang | 22.60409 | 113.10194 |
| 304 | Jiangmen | Peng Jiang Qu Ba Chong Shi Chang | 22.61253 | 113.08452 |
| 305 | Jiangmen | Peng Jiang Qu Du Ruan Zhen Guan Xi Shi Chang | 22.59499 | 113.07192 |
| 306 | Jiangmen | Sha Ping Jie Dao Xin Hua Shi Chang | 22.77106 | 112.96864 |
| 307 | Jiangmen | Sha Tang Shi Chang | 22.45647 | 112.59614 |
| 308 | Jiangmen | Yang Qiao Shi Chang | 22.58014 | 113.09052 |
| 309 | Jiangmen | Yu De Shi Chang | 22.61405 | 113.09227 |
| 310 | Jiangmen | Zhao Kang Shi Chang | 22.51401 | 113.04293 |
| 311 | Jieyang | Bai Ta Shi Chang | 23.56739 | 116.21448 |
| 312 | Jieyang | Cheng Bei Shi Chang | 23.31290 | 116.17157 |
| 313 | Jieyang | Chi Wei Shi Chang | 23.30673 | 116.13863 |
| 314 | Jieyang | Gou Kou Shi Chang | 23.55700 | 116.38524 |
| 315 | Jieyang | He Po Da Tong Shi Chang | 23.43468 | 115.84646 |
| 316 | Jieyang | Hui Xi Shi Chang | 23.03516 | 116.29346 |
| 317 | Jieyang | Hui Zhai Shi Chang | 23.48865 | 116.01358 |
| 318 | Jieyang | Jie Dong Qu Cheng Xi Shi Chang | 23.54963 | 116.33209 |
| 319 | Jieyang | Jie Xi He Dong Fu Shi Shi Chang | 23.43578 | 115.85293 |
| 320 | Jieyang | Jin He Zhen Shi Chang | 23.43061 | 116.05845 |
| 321 | Jieyang | Kong Gang Qu Gang Kou Shi Chang | 23.51947 | 116.42823 |
| 322 | Jieyang | Kong Gang Qu Yu Jiang Shi Chang | 23.52260 | 116.42334 |
| 323 | Jieyang | Kui Dong Shi Chang | 23.03660 | 116.30522 |
| 324 | Jieyang | Kui Tan Xin Xing Shi Chang | 23.56892 | 116.39597 |
| 325 | Jieyang | Liu Sha Dong Shi Chang | 23.30126 | 116.18339 |
| 326 | Jieyang | Long Jiang Lin Shi Shi Chang | 22.99081 | 116.18636 |
| 327 | Jieyang | Ma Ya Shi Chang | 23.55483 | 116.36785 |
| 328 | Jieyang | Mei Yun Qiao Tou | 23.52969 | 116.29511 |
| 329 | Jieyang | Pao Tai Han Hua Shi Chang | 23.51268 | 116.49293 |
| 330 | Jieyang | Peng Lai Shi Chang | 23.03420 | 116.30399 |
| 331 | Jieyang | Pu Ning Shi Dong Shi Chang | 23.30101 | 116.18504 |
| 332 | Jieyang | Qiao Lin Qiao Tou | 23.55964 | 116.34167 |
| 333 | Jieyang | Tan Cai Cun Shi Chang | 23.56579 | 116.29680 |
| 334 | Jieyang | Tong Pan Chi Shi Chang | 23.04181 | 116.29825 |
| 335 | Jieyang | Wu Jing Fu Zhen Shi Chang | 23.56898 | 116.07944 |
| 336 | Jieyang | Xin He Shi Chang | 23.56173 | 116.38356 |
| 337 | Jieyang | Yang Mei Shi Chang | 23.02911 | 116.29796 |
| 338 | Jieyang | Zuo Hua Shi Chang | 23.54043 | 116.37925 |
| 339 | Jieyang | Zuo Xi Shi Chang | 23.54988 | 116.33885 |
| 340 | Maoming | Fu Hua Nong Mao Shi Chang | 21.67238 | 110.94072 |
| 341 | Maoming | He Xi Nong Mao Shi Chang | 21.67404 | 110.90711 |
| 342 | Maoming | Hua Zhou Shi Bei An San Niao Pi Fa Shi Chang | 21.64035 | 110.66479 |
| 343 | Maoming | Hua Zhou Shi Zuo Zhou Shi Chang | 21.65793 | 110.65031 |
| 344 | Maoming | Mao Nan Qu Chao Yang Shi Chang | 21.65620 | 110.92266 |
| 345 | Maoming | Mao Nan Qu He Dong Zong He Shi Chang | 21.66505 | 110.92355 |
| 346 | Maoming | Mao Nan Qu Zuo Hua San Niao Pi Fa Shi Chang | 21.59031 | 110.94172 |
| 347 | Maoming | Mao Nan Shan Ge San Niao Pi Fa Shi Chang | 21.72820 | 110.94165 |
| 348 | Maoming | Shan Ge Zhen San Niao Pi Fa Shi Chang | 21.72801 | 110.94203 |
| 349 | Meizhou | Bai Hua Zhou Shi Chang | 24.30491 | 116.12865 |
| 350 | Meizhou | Chang Xing Lu Dong Fu Jie | 24.65553 | 116.17641 |
| 351 | Meizhou | Cheng Jiang Gang Zi Shang Shi Chang | 24.28143 | 116.09946 |
| 352 | Meizhou | Dong Feng Shi Chang | 24.35653 | 116.70435 |
| 353 | Meizhou | Dong Xiang Shi Chang | 24.32676 | 116.12902 |
| 354 | Meizhou | Hong Xing San Niao Pi Fa Shi Chang | 24.33263 | 116.10562 |
| 355 | Meizhou | Hua Jian Shi Chang | 24.29005 | 116.13000 |
| 356 | Meizhou | Huang Zhu Zuo | 24.15245 | 115.72272 |
| 357 | Meizhou | Lou Xia Tang Shi Chang | 24.32279 | 116.12754 |
| 358 | Meizhou | Mei Xian Bai Du San Niao Pi Fa Shi Chang | 24.45752 | 116.17769 |
| 359 | Meizhou | Nan Qu Shi Chang | 24.57492 | 115.89954 |
| 360 | Meizhou | San Jiao Zhong Xin Shi Chang | 24.27312 | 116.11923 |
| 361 | Meizhou | Shui Zhai Zhen Da Ba Shi Chang | 23.94800 | 115.78070 |
| 362 | Meizhou | Tang Keng Zhen Nan Shi Chang | 23.76369 | 116.18785 |
| 363 | Meizhou | Tang Keng Zhen Shang Hu Lu | 23.75473 | 116.19444 |
| 364 | Meizhou | Tang Keng Zhen Zhan Qian Lu | 23.75586 | 116.19745 |
| 365 | Qingyuan | Qing Cheng Qu Hou Gang Pi Fa Shi Chang | 23.72793 | 113.03743 |
| 366 | Qingyuan | Qing Cheng Qu Nan Shi Chang | 23.69854 | 113.04357 |
| 367 | Qingyuan | Qing Cheng Qu Qu Keng Kou Shi Chang | 23.71077 | 113.04262 |
| 368 | Qingyuan | Qing Cheng Qu Xia Kuo Shi Chang | 23.70819 | 113.02988 |
| 369 | Qingyuan | Qing Cheng Qu Xin Cheng Nan Shi Chang | 23.69850 | 113.04366 |
| 370 | Qingyuan | Qing Cheng Qu Yi Tai Shi Chang | 23.71934 | 113.03949 |
| 371 | Qingyuan | Qing Xin Qu Qing He Rou Cai Shi Chang | 23.73879 | 113.01343 |
| 372 | Qingyuan | Qing Xin Qu Qing He Shi Chang | 23.73694 | 113.02365 |
| 373 | Qingyuan | Qing Xin Qu Tai He Zhen Ming Xia Shi Chang | 23.74540 | 113.01718 |
| 374 | Qingyuan | Qing Xin Qu Tai He Zhen Tai He Zong He Shi Chang | 23.73966 | 113.02008 |
| 375 | Qingyuan | Qing Xin Qu Zhen Xing Shi Chang | 23.73414 | 113.01091 |
| 376 | Qingyuan | Qing Xin Tai He Rou Cai Shi Chang | 23.73990 | 113.02076 |
| 377 | Qingyuan | Xiang Qun Rou Cai Shi Chang | 23.73770 | 113.02541 |
| 378 | Qingyuan | Xin Cheng San Jiao Shi Chang | 23.68327 | 113.05900 |
| 379 | Shantou | Ba Tou Tu Chi Zong He Shi Chang | 23.44446 | 116.83759 |
| 380 | Shantou | Chai Jing Huo Ji Zhuan Shou Dian | 23.45180 | 116.84706 |
| 381 | Shantou | Chang Xia Shi Chang | 23.36318 | 116.71162 |
| 382 | Shantou | Chao Nan Qu Xia Shan Jie Dao Nan Xing Shi Chang | 23.25445 | 116.43520 |
| 383 | Shantou | Chao Yang Qu Wen Guang Dong Men Shi Chang | 23.26491 | 116.60683 |
| 384 | Shantou | Dong Men Shi Chang | 23.39994 | 116.71871 |
| 385 | Shantou | Fu He Shi Chang | 23.36256 | 116.68143 |
| 386 | Shantou | Hou Zhai Zhen Zhong Xin Shi Chang | 23.42739 | 117.03272 |
| 387 | Shantou | Jin Ping Qu Guang Xia Xin Cheng Zong He Shi Chang | 23.39944 | 116.71828 |
| 388 | Shantou | Jin Tai Shi Chang | 23.36289 | 116.75170 |
| 389 | Shantou | Long Hu Qu Xin Xi Zhen Zhong San He Shi Chang | 23.40176 | 116.78221 |
| 390 | Shantou | Long Hu Shi Chang | 23.44467 | 116.76054 |
| 391 | Shantou | Long Yan Shi Chang | 23.36955 | 116.71747 |
| 392 | Shantou | Nan Men Shi Chang | 23.25735 | 116.60764 |
| 393 | Shantou | Ning Guan Yuan Shi Chang | 23.47610 | 116.76115 |
| 394 | Shantou | Ou Ting Shi Chang | 23.41244 | 116.72239 |
| 395 | Shantou | Ping Yuan Shi Chang | 23.36911 | 116.70920 |
| 396 | Shantou | Xia Shan Xi Gou Shi Chang | 23.25466 | 116.41982 |
| 397 | Shantou | Xin Xi Shi Chang | 23.46960 | 116.77741 |
| 398 | Shantou | Ya Qian Shi Chang | 23.46319 | 116.77588 |
| 399 | Shantou | Zhong Shan Dong Fu Shi Pin Zong He Shi Chang | 23.36401 | 116.71698 |
| 400 | Shantou | Zhong Shan Dong Shi Chang | 23.36369 | 116.71671 |
| 401 | Shantou | Zhu He Zong He Shi Chang | 23.37914 | 116.73758 |
| 402 | Shantou | Zuo Jiang Qu Chi Gang Dong Men Shi Chang | 23.28726 | 116.73816 |
| 403 | Shantou | Zuo Jiang Shi Chang | 23.28610 | 116.72622 |
| 404 | Shanwei | Wen Shi Qin Lei Pi Fa Shi Chang | 22.79445 | 115.37118 |
| 405 | Shaoguan | Qian Jin Shi Chang | 24.80435 | 113.57775 |
| 406 | Shaoguan | San Niao Pi Fa Shi Chang | 24.77808 | 113.60217 |
| 407 | Shenzhen | Bao An Qu Fu Yong Jie Dao Qiao Tou Shi Chang | 22.67416 | 113.82106 |
| 408 | Shenzhen | Bao An Qu Sha Jing Jie Dao Ming Zhu Shi Chang | 22.74172 | 113.81849 |
| 409 | Shenzhen | Bao An Qu Shi Yan Jie Dao Shi Yan Shi Chang | 22.67979 | 113.93032 |
| 410 | Shenzhen | Bao An Qu Song Gang Jie Dao Zuo Cui Shi Chang | 22.77297 | 113.84996 |
| 411 | Shenzhen | Bao An Qu Xi Xiang Jie Dao Long Zhu Shi Chang | 22.58673 | 113.88518 |
| 412 | Shenzhen | Da Peng Xin Qu Kui Yong Lao Shi Chang | 22.63985 | 114.42722 |
| 413 | Shenzhen | Da Peng Xin Qu Wang Mu Shi Chang | 22.60230 | 114.48322 |
| 414 | Shenzhen | Fu Tian Qu Huang Gang Shi Chang | 22.53898 | 114.07724 |
| 415 | Shenzhen | Fu Tian Qu Nong Pi Shi Chang | 22.56821 | 114.04455 |
| 416 | Shenzhen | Fu Tian Qu Shui Wei Shi Chang | 22.52516 | 114.07073 |
| 417 | Shenzhen | Guang Ming Xin Qu Gong Ming Zong He Shi Chang | 22.78680 | 113.89954 |
| 418 | Shenzhen | Guang Ming Xin Qu Guang Ming Shi Chang | 22.76332 | 113.95835 |
| 419 | Shenzhen | Guang Ming Xin Qu Tian Zuo Shi Chang | 22.73170 | 113.91002 |
| 420 | Shenzhen | Heng Gang Jie Dao Pai Bang Shi Chang | 22.65651 | 114.20498 |
| 421 | Shenzhen | Long Gang Nan Ling Wan Jia Le Chao Shi | 22.61672 | 114.15736 |
| 422 | Shenzhen | Long Gang Qu Bu Ji Hong Zuo Shi Chang | 22.64016 | 114.10668 |
| 423 | Shenzhen | Long Gang Qu Heng Gang Shi Chang Jia Qin Dang | 22.65686 | 114.20315 |
| 424 | Shenzhen | Long Gang Qu Heng Gang Si Lian Pai Bang Shi Chang | 22.65670 | 114.20516 |
| 425 | Shenzhen | Long Gang Qu Nan Wan Jie Dao Kang Qiao Shi Chang | 22.62960 | 114.14941 |
| 426 | Shenzhen | Long Gang Qu Ping Di Jie Dao Min Xin Shi Chang | 22.77930 | 114.31700 |
| 427 | Shenzhen | Long Gang Qu Ping Di Jie Dao Yue Hu Gang Shi Chang | 22.77855 | 114.31187 |
| 428 | Shenzhen | Long Gang Qu Ping Hu Jie Dao Ping Hu Shi Chang | 22.70806 | 114.13235 |
| 429 | Shenzhen | Guan Lan Jin Li Nong Mao Shi Chang | 22.70844 | 114.05365 |
| 430 | Shenzhen | Long Hua Xin Qu Guan Lan Niu Hu Shi Chang | 22.72376 | 114.10635 |
| 431 | Shenzhen | L Lan Zhang Keng Jing Shi Chang | 22.69250 | 114.08452 |
| 432 | Shenzhen | Long Hua Xin Qu Guan Lan Zong He Shi Chang | 22.72654 | 114.06853 |
| 433 | Shenzhen | Luo Hu Qu Bu Xin Shi Chang | 22.58276 | 114.13559 |
| 434 | Shenzhen | Luo Hu Qu Dong Men Shi Chang | 22.55253 | 114.13062 |
| 435 | Shenzhen | Luo Hu Qu Feng Hu Nong Pi Cui Yuan | 22.58286 | 114.12674 |
| 436 | Shenzhen | Luo Hu Qu He Hua Shi Chang | 22.56102 | 114.14589 |
| 437 | Shenzhen | Nan Shan Jia Pi Jia Qin Pi Fa Shi Chang | 22.56457 | 113.92977 |
| 438 | Shenzhen | Nan Shan Qu She Kou Shi Chang | 22.49202 | 113.93490 |
| 439 | Shenzhen | Nan Shan Qu Wan Xia Cun Wei Shi Chang | 22.49444 | 113.93977 |
| 440 | Shenzhen | Nan Shan San Niao Shi Chang | 22.57223 | 113.94622 |
| 441 | Shenzhen | Ping Shan Xin Qu Keng Zuo Di Er Shi Chang | 22.75302 | 114.38520 |
| 442 | Shenzhen | Ping Shan Xin Qu Ping Shan Zong He Shi Chang | 22.69581 | 114.34612 |
| 443 | Shenzhen | Qun Xing Nong Mao Shi Chang | 22.66501 | 114.02382 |
| 444 | Shenzhen | Yan Tian Qu Ming Zhu Shi Chang | 22.59523 | 114.26733 |
| 445 | Shenzhen | Yan Tian Qu Tian Xin Shi Chang | 22.55673 | 114.23719 |
| 446 | Foshan | Lun Jiao San Zhou Nong Chan Pin Jiao Yi Zhong Xin | 22.88554 | 113.27965 |
| 447 | Foshan | Shun De Qu Da Liang Fu You Shi Chang | 22.83901 | 113.27052 |
| 448 | Foshan | Shun De Qu Da Liang Xian Dai Jie Shi | 22.83791 | 113.28025 |
| 449 | Foshan | Shun De Qu Rong Gui Hua Feng Shi Chang | 22.75808 | 113.26333 |
| 450 | Yangjiang | Bu Chang Zuo Shi Chang | 21.79442 | 111.94761 |
| 451 | Yangjiang | Cheng Nan Shi Chang | 22.16821 | 111.79485 |
| 452 | Yangjiang | Di Er Shi Chang | 22.16889 | 111.80300 |
| 453 | Yangjiang | Dong Cheng Dong Xing Shi Chang | 21.87288 | 112.00971 |
| 454 | Yangjiang | Dong Cheng Jin Cun Shi Chang | 21.89697 | 112.03318 |
| 455 | Yangjiang | Dong Cheng Zhen Chang Long Shi Chang | 21.86902 | 112.02328 |
| 456 | Yangjiang | Dong Cheng Zhen Chang Xing Shi Chang | 21.89695 | 112.03305 |
| 457 | Yangjiang | Dong Cheng Zhen Jin Cun Shi Chang | 21.89423 | 112.02795 |
| 458 | Yangjiang | Dong Hu Shi Chang | 21.86905 | 111.99321 |
| 459 | Yangjiang | Dong Men Shi Chang | 22.17437 | 111.80161 |
| 460 | Yangjiang | Er Huan Shi Chang | 21.85861 | 111.98220 |
| 461 | Yangjiang | Hai Ling Bei Ji Cun Shi Chang | 21.57793 | 111.84835 |
| 462 | Yangjiang | Hai Ling Shi Chang | 21.65897 | 111.96717 |
| 463 | Yangjiang | He Shan He Ti Shi Chang | 21.96646 | 112.13865 |
| 464 | Yangjiang | He Shan Zhen Zhu Zi Lie Shi Chang | 21.96982 | 112.13732 |
| 465 | Yangjiang | He Xi Lian Ping Shi Chang | 22.18628 | 111.77495 |
| 466 | Yangjiang | He Xi San Niao Pi Fa Shi Chang | 22.18569 | 111.78929 |
| 467 | Yangjiang | Hua Hong Rou Cai Shi Chang | 21.87186 | 112.00959 |
| 468 | Yangjiang | Jiang Cheng Qu Cheng Xi Shi Chang | 21.85374 | 111.95443 |
| 469 | Yangjiang | Jiang Cheng Qu Di Yi Shi Chang | 21.85168 | 111.96366 |
| 470 | Yangjiang | Jiang Cheng Qu Dong Yuan Shi Chang | 21.86810 | 111.97602 |
| 471 | Yangjiang | Jiang Cheng Qu Gang Lie Shi Chang | 21.85315 | 111.97256 |
| 472 | Yangjiang | Jiang Cheng Qu Jin Ji Shi Chang | 21.86181 | 111.96193 |
| 473 | Yangjiang | Jiang Cheng Qu Mu Zuo Shi Chang | 21.84699 | 111.98805 |
| 474 | Yangjiang | Jiang Cheng Qu San Niao Pi Fa Shi Chang | 21.88494 | 111.94177 |
| 475 | Yangjiang | Jiang Cheng Qu Shi Zi Shan Shi Chang | 21.86277 | 111.98279 |
| 476 | Yangjiang | Ling Dong Shi Chang | 21.86085 | 112.01480 |
| 477 | Yangjiang | Ma Yan Qin Niao Pi Fa Shi Chang | 21.88521 | 111.93784 |
| 478 | Yangjiang | Mo Yang Shi Chang | 22.17380 | 111.78718 |
| 479 | Yangjiang | Pu Pai Shi Chang | 21.68682 | 111.55023 |
| 480 | Yangjiang | Ru Dong Chang Li Shi Chang | 21.60802 | 111.43996 |
| 481 | Yangjiang | Ru Dong Shi Chang | 21.60201 | 111.44094 |
| 482 | Yangjiang | Tang Kou Shi Chang | 21.83268 | 111.58739 |
| 483 | Yangjiang | Xian Cheng Xin Cheng Shi Chang | 21.75719 | 111.61937 |
| 484 | Yangjiang | Xing Yuan Shi Chang | 21.86964 | 111.96368 |
| 485 | Yangjiang | Ya Shao Zhen Shi Chang | 21.82643 | 112.05880 |
| 486 | Yangjiang | Yang Chun Shi Cheng Bei Shi Chang | 22.18314 | 111.80584 |
| 487 | Yangjiang | Yang Chun Shi He Xi San Niao Shi Chang | 22.18845 | 111.77575 |
| 488 | Yangjiang | Yang Dong Dong Cheng Da Ling Shi Chang | 21.89658 | 112.03442 |
| 489 | Yangjiang | Yang Dong Xian Dong Run Shi Chang | 21.86131 | 112.01565 |
| 490 | Yangjiang | Yang Dong Xian Dong Xing Shi Chang | 21.87273 | 112.00997 |
| 491 | Yangjiang | Yang Dong Xian Shi Xing Shi Chang | 21.86313 | 112.01204 |
| 492 | Yangjiang | Yang Xi Xian Cheng Cun Zhen Shi Chang | 21.80986 | 111.72784 |
| 493 | Yangjiang | Yang Xi Xian Dong Hu Nong Mao Shi Chang | 21.75328 | 111.63298 |
| 494 | Yangjiang | Yang Xi Xian Shi Si Qu Shi Chang | 21.75686 | 111.61961 |
| 495 | Yangjiang | Yang Xi Xian Zhi Gong Zhen Di Er Shi Chang | 21.75283 | 111.62123 |
| 496 | Yangjiang | Yang Xi Yong Fu Shi Chang | 21.76941 | 111.62857 |
| 497 | Yangjiang | Yang Xi Zhi Gong Zhen An He Shi Chang | 21.74755 | 111.62120 |
| 498 | Yangjiang | Yin Wan Shi Chang | 21.88015 | 111.97464 |
| 499 | Yangjiang | Zha Po Nong Mao Shi Chang | 21.57789 | 111.83821 |
| 500 | Yunfu | Cha Ting Shi Chang | 22.76821 | 111.58549 |
| 501 | Yunfu | Dian Xin Dui Mian Shi Chang | 23.15640 | 111.48287 |
| 502 | Yunfu | Du Cheng Bei Jiao Shi Chang | 23.24890 | 111.53742 |
| 503 | Yunfu | Gao Feng Zhen Shi Ti Zuo Ji Chang | 22.96041 | 112.04743 |
| 504 | Yunfu | Liu Du Shi Chang | 23.08217 | 112.01555 |
| 505 | Yunfu | Luo Cheng Zhen Ping Nan Shi Chang | 22.77466 | 111.57717 |
| 506 | Yunfu | Luo Gui Qiao Shi Chang | 22.95371 | 112.05011 |
| 507 | Yunfu | Xi Jie Shi Chang | 22.70356 | 112.23265 |
| 508 | Yunfu | Xia Dong Shi Chang | 22.94231 | 112.19964 |
| 509 | Yunfu | Xin Xing Cheng Nan Shi Chang | 22.69599 | 112.23654 |
| 510 | Yunfu | Xin Xing Xian Xin Feng Rou Cai Shi Chang | 22.69447 | 112.23795 |
| 511 | Yunfu | Xin Xing Xian Xin Feng Shi Chang | 22.70211 | 112.23956 |
| 512 | Yunfu | Yu Nan Lian Tan Shi Chang | 22.92811 | 111.73196 |
| 513 | Yunfu | Yu Nan Xian Du Cheng Shi Chang | 23.24914 | 111.53721 |
| 514 | Yunfu | Yun Cheng Qu Long Hua Lu Shi Chang | 22.92445 | 112.04273 |
| 515 | Yunfu | Yun Cheng Qu Wen Shi Si Lao Ya Chang | 22.92449 | 112.23636 |
| 516 | Yunfu | Yun Fu Mu Yang Luo Shi Dan Ji Chang | 22.95974 | 112.06535 |
| 517 | Zhanjiang | Chi Kan Bai Yuan Shi Chang | 21.27308 | 110.37708 |
| 518 | Zhanjiang | Chi Kan Qu Bai Yuan Shi Chang | 21.27377 | 110.37765 |
| 519 | Zhanjiang | Dong Zuo Shi Chang | 21.71509 | 110.09299 |
| 520 | Zhanjiang | Gao Tian San Niao Pi Fa Shi Chang | 21.51120 | 110.31791 |
| 521 | Zhanjiang | Lian Jiang Shi Gao Tian Shi Chang | 21.89256 | 110.11964 |
| 522 | Zhanjiang | Huang Wai Nong Fu Chan Pin Mao Yi Shi Chang | 21.27409 | 110.33231 |
| 523 | Zhanjiang | Ma Zhang Shi Chang | 21.27256 | 110.33470 |
| 524 | Zhanjiang | Mei Lu Zhong Xin Shi Chang | 21.42181 | 110.78354 |
| 525 | Zhanjiang | Po Tou Qu Hai Dong Shi Chang | 21.25088 | 110.46116 |
| 526 | Zhanjiang | Po Tou Qu Long Tou Shi Chang | 21.36319 | 110.52798 |
| 527 | Zhanjiang | Sui Xi Xian Sui Cheng Zhen Zhong Xin Shi Chang | 21.38499 | 110.25902 |
| 528 | Zhanjiang | Xi Hu Zong He Shi Chang | 21.27080 | 110.37312 |
| 529 | Zhanjiang | Xia Shan Bu Xing Jie Rou Cai Shi Chang | 21.20731 | 110.41458 |
| 530 | Zhanjiang | Xia Shan Gong Nong Shi Chang | 21.19032 | 110.40809 |
| 531 | Zhanjiang | Xia Shan Qu Dong Shan San Niao Shi Chang | 21.03770 | 110.39303 |
| 532 | Zhanjiang | Xia Shan Qu Fang Xing Shi Chang | 21.21440 | 110.39882 |
| 533 | Zhanjiang | Xia Shan Qu Lu Lin Shi Chang | 21.21364 | 110.40501 |
| 534 | Zhanjiang | Xia Shan Qu Xing Long San Niao Pi Fa Shi Chang | 21.19084 | 110.40204 |
| 535 | Zhanjiang | Xu Wen Xian Min Kang Shi Chang | 20.33166 | 110.17566, |
| 536 | Zhanjiang | Zuo Xin Shi Chang | 21.62093 | 110.28546 |
| 537 | Zhaoqing | Cang Feng San Niao Pi Fa Shi Chang | 23.36050 | 112.67253 |
| 538 | Zhaoqing | Da Gang Zhen Shi Chang | 23.89368 | 112.05208 |
| 539 | Zhaoqing | De Qing De Cheng Kang Da Shi Chang | 23.14789 | 111.77714 |
| 540 | Zhaoqing | Di Dou Shi Chang | 23.57171 | 112.72468 |
| 541 | Zhaoqing | Duan Zhou Qu Mu Gang San Niao Pi Fa Shi Chang | 23.07456 | 112.43287 |
| 542 | Zhaoqing | Duan Zhou Qu Zhong Xin Shi Chang | 23.05378 | 112.47748 |
| 543 | Zhaoqing | Feng Gang Shi Chang | 23.09062 | 112.52269 |
| 544 | Zhaoqing | Feng Gang Zhen Shi Chang | 24.00282 | 112.36367 |
| 545 | Zhaoqing | Feng Kai Chang An Rou Cai Shi Chang | 23.88883 | 111.90699 |
| 546 | Zhaoqing | Feng Kai Jiang Kou Da Tang Shi Chang | 23.44550 | 111.50678 |
| 547 | Zhaoqing | Feng Kai Jiang Kou Zhong Xin Shi Chang | 23.44030 | 111.50968 |
| 548 | Zhaoqing | Feng Kai Nan Feng Zhong Xin Shi Chang | 23.74891 | 111.80863 |
| 549 | Zhaoqing | Gan Sa Zhen Shi Chang | 23.95805 | 112.34152 |
| 550 | Zhaoqing | Gang Ping Zhen Shi Chang | 23.99432 | 111.98164 |
| 551 | Zhaoqing | Guang Ning San Niao Pi Fa Shi Chang | 23.72981 | 112.63529 |
| 552 | Zhaoqing | Gui Cheng Keng Kou Shi Chang | 23.16213 | 112.56943 |
| 553 | Zhaoqing | Hua Sheng Shi Chang | 23.31179 | 112.82684 |
| 554 | Zhaoqing | Huai Cheng Cheng Xi Shi Chang | 23.91750 | 112.18786 |
| 555 | Zhaoqing | Huai Cheng Guo Mao San Niao Shi Chang | 23.92327 | 112.20522 |
| 556 | Zhaoqing | Huai Cheng Jiang Bin Rou Cai Shi Chang | 23.91318 | 112.19073 |
| 557 | Zhaoqing | Huai Cheng Yan Jiang Shi Chang | 23.91339 | 112.19242 |
| 558 | Zhaoqing | Huang Gang Zhen Shi Chang | 23.05545 | 112.49525 |
| 559 | Zhaoqing | Jiang Gu Shi Chang | 23.50867 | 112.66499 |
| 560 | Zhaoqing | Jin Du Shi Chang | 23.04717 | 112.52457 |
| 561 | Zhaoqing | Lan Zhong Zhen Shi Chang | 24.08108 | 111.96662 |
| 562 | Zhaoqing | Leng Keng Zhen Quan You San Niao Pi Fa Bu | 24.04068 | 112.05434 |
| 563 | Zhaoqing | Leng Keng Zhen Shi Chang | 24.04093 | 112.05242 |
| 564 | Zhaoqing | Lian Mai Zhen Shi Chang | 24.05586 | 112.17225 |
| 565 | Zhaoqing | Liang Cun Zhen San Niao Pi Fa Shi Chang | 24.05456 | 112.17226 |
| 566 | Zhaoqing | Liang Cun Zhen Shi Chang | 23.95334 | 112.03453 |
| 567 | Zhaoqing | Ma An Shi Chang | 22.99820 | 112.43067 |
| 568 | Zhaoqing | Ma Ning Zhen Shi Chang | 24.03428 | 111.99052 |
| 569 | Zhaoqing | Ma Tian Shi Chang | 23.34696 | 112.71125 |
| 570 | Zhaoqing | Nan An Zhong Xin Shi Chang | 23.03202 | 112.46619 |
| 571 | Zhaoqing | Nan Jie Pan He Wan Shi Chang | 23.63495 | 112.43683 |
| 572 | Zhaoqing | Nan Jie Shi Chang | 23.64157 | 112.44541 |
| 573 | Zhaoqing | Nan Jie Wu Yi Da Shi Chang | 23.63543 | 112.44921 |
| 574 | Zhaoqing | Ning Feng Jia Qin Shui Chan Gong Si | 23.63562 | 112.45395 |
| 575 | Zhaoqing | Qia Shui Zhen Shi Chang | 24.09239 | 112.35123 |
| 576 | Zhaoqing | Qiao Tou Zhen Shi Chang | 23.75839 | 111.97870 |
| 577 | Zhaoqing | Shang Tang Jie Shi Chang | 23.90923 | 112.19177 |
| 578 | Zhaoqing | Shi Dong Shi Chang | 23.62328 | 112.08042 |
| 579 | Zhaoqing | Shui Keng Yi Shi Chang | 23.19842 | 112.58271 |
| 580 | Zhaoqing | Si Hui Gao Guan Shi Chang | 23.34530 | 112.70113 |
| 581 | Zhaoqing | Si Hui San Niao Pi Fa Shi Chang | 23.36586 | 112.69267 |
| 582 | Zhaoqing | Si Hui Zuo Shan Shi Chang | 23.34897 | 112.70230 |
| 583 | Zhaoqing | Xia Shuai Zhen Shi Chang | 24.21770 | 112.09645 |
| 584 | Zhaoqing | Xiang Jiang Shi Chang | 23.30400 | 112.81797 |
| 585 | Zhaoqing | Xiao Xiang Yang Guang Ji Chang | 23.14025 | 112.40630 |
| 586 | Zhaoqing | Xin Qiao Shi Chang | 22.95349 | 112.42324 |
| 587 | Zhaoqing | Yong Gu Zhen Shi Chang | 23.72476 | 112.08384 |
| 588 | Zhaoqing | Zha Gang Zhen Shi Chang | 23.84448 | 112.11506 |
| 589 | Zhaoqing | Zhao Qing Gao Yao Nan An Cheng Dong Shi Chang | 23.03321 | 112.46674 |
| 590 | Zhaoqing | Zhong Zhou Zhen Shi Chang | 24.11856 | 112.17154 |
| 591 | Zhaoqing | Zuo Lang Zhen Shi Chang | 23.99557 | 112.29845 |
| 592 | Zhaoqing | Zuo Zi Zhen Shi Chang | 23.79690 | 112.30581 |
| 593 | Zhongshan | Ai Guo Shi Chang | 22.68180 | 113.43530 |
| 594 | Zhongshan | An Shan Shi Chang | 22.51349 | 113.36581 |
| 595 | Zhongshan | Dong Nan Men Shi Chang | 22.52687 | 113.38393 |
| 596 | Zhongshan | Dong Sheng Shi Chang | 22.54739 | 113.39758 |
| 597 | Zhongshan | Gao Ya Shi Chang | 22.70608 | 113.47405 |
| 598 | Zhongshan | Gu Si Shi Chang | 22.62304 | 113.18378 |
| 599 | Zhongshan | Guang Ming Shi Chang | 22.53199 | 113.37560 |
| 600 | Zhongshan | Hai Bang Xi Jie Qin Lei Jia Gong Chang | 22.59460 | 113.38275 |
| 601 | Zhongshan | Hou Xing Shi Chang | 22.53756 | 113.40653 |
| 602 | Zhongshan | Jie Min Shang Ye Guang Chang | 22.68264 | 113.41777 |
| 603 | Zhongshan | Jie Min Shi Chang | 22.68319 | 113.41906 |
| 604 | Zhongshan | Ku Chong Shi Chang | 22.51718 | 113.40104 |
| 605 | Zhongshan | Min Zu Dong Shi Chang | 22.52523 | 113.38458 |
| 606 | Zhongshan | Nan Xia Shi Chang | 22.51293 | 113.38596 |
| 607 | Zhongshan | San Jiao Shi Chang | 22.69101 | 113.43939 |
| 608 | Zhongshan | San Niao Shi Chang | 22.59890 | 113.31767 |
| 609 | Zhongshan | Sha Lan Shi Chang | 22.67401 | 113.41242 |
| 610 | Zhongshan | Tai An Shi Chang | 22.52476 | 113.37434 |
| 611 | Zhongshan | Yan Ling Shi Chang | 22.53307 | 113.38180 |
| 612 | Zhongshan | Yuan Feng Shi Chang | 22.54151 | 113.37900 |
| 613 | Zhongshan | Zhong Xin Shi Chang | 22.45995 | 113.35150 |
| 614 | Zhuhai | Bai Jiao Da Zhong Shi Chang | 22.21823 | 113.31273 |
| 615 | Zhuhai | Bai Jiao San Niao Pi Fa Shi Chang | 22.21897 | 113.32174 |
| 616 | Zhuhai | Chao Yang Shi Chang | 22.28544 | 113.58516 |
| 617 | Zhuhai | Dou Men Qu Jing An Zhen Tian Yang Shi Chang | 22.21507 | 113.30216 |
| 618 | Zhuhai | He Ping San Niao Pi Fa Shi Chang | 22.30295 | 113.49683 |
| 619 | Zhuhai | Ji Lian Shi Chang | 22.25212 | 113.57735 |
| 620 | Zhuhai | Jing An Bei Ao Shi Chang | 22.22899 | 113.29589 |
| 621 | Zhuhai | Jing An Nan Chao Shi Chang | 22.20513 | 113.30399 |
| 622 | Zhuhai | Jing An Zuo Wan Shi Chang | 22.20225 | 113.30574 |
| 623 | Zhuhai | Nan Chao Shi Chang | 22.28200 | 113.57280 |
| 624 | Zhuhai | Nan Keng Shi Chang | 22.28210 | 113.57283 |
| 625 | Zhuhai | San Zao Tang Ren Jie Shi Chang | 22.05595 | 113.35380 |
